# The Role of Extracellular Vesicles in the Pathogenesis and Treatment of Autoimmune Disorders

**DOI:** 10.3389/fimmu.2021.566299

**Published:** 2021-02-24

**Authors:** Mengrou Lu, Emma DiBernardo, Emily Parks, Hannah Fox, Si-Yang Zheng, Elizabeth Wayne

**Affiliations:** ^1^ Department of Electrical and Computer Engineering, College of Engineering, Carnegie Mellon University, Pittsburgh, PA, United States; ^2^ Department of Chemical Engineering, College of Engineering, Carnegie Mellon University, Pittsburgh, PA, United States

**Keywords:** extracellular vesicle, autoimmunity, antigen presentation, immune-related adverse events, therapeutic delivery

## Abstract

Extracellular vesicles (EVs) are important players in autoimmune diseases, both in disease pathogenesis and as potential treatments. EVs can transport autoimmune triggers throughout the body, facilitating the process of antigen presentation. Understanding the link between cellular stress and EV biogenesis and intercellular trafficking will advance our understanding of autoimmune diseases. In addition, EVs can also be effective treatments for autoimmune diseases. The diversity of cell types that produce EVs leads to a wide range of molecules to be present in EVs, and thus EVs have a wide range of physiological effects. EVs derived from dendritic cells or mesenchymal stem cells have been shown to reduce inflammation. Since many autoimmune treatments are focused only on symptom management, EVs present a promising avenue for potential treatments. This review looks at the different roles EVs can play in autoimmune diseases, from disease pathology to diagnosis and treatment. We also overview various methodologies in isolating or generating EVs and look to the future for possible applications of EVs in autoimmune diseases.

## Introduction to Extracellular Vesicle (EV) Biology

Our understanding of the role of extracellular vesicles (EVs) in biological processes is rapidly evolving. There is substantial literature connecting cellular stress to autoimmunity, but surprisingly there are few that conceptualize EVs as the communication link (1–3). When EVs were first discovered, they were believed to be a final product, a mechanism for discarding unnecessary cellular materials (4). Subsequent studies showed that EVs could also function to transmit instructions for cell migration, proliferation, and differentiation (5). Later on, the role of EVs modulating immune responses through antigen presentation was uncovered (6). Immune cells secrete EVs and also receive cellular instructions from EVs that guide further immune reactions (7). We believe this is an emerging trend that should be evaluated when considering diagnostic and therapeutic applications.

EVs are produced by a variety of cells, and their content resembles their host cells—the lipid composition of the outer cell membrane makes up the outer membrane of the EV. In addition, EVs contain a mixture of bioactive molecules—i.e. proteins, nucleic acids, metabolites—derived from the cytoplasmic content of the host cell. Importantly, however, the concentration may not be similar, and studies have shown enrichment of proteins in EVs that was not apparent from the host cells (6, 8).

While exosome is the classical term, EV has become the more general term to encapsulate both the diversity and complexity of vesicles observed (9, 10). EVs range in size from 30 nm nanovesicles to ectosomes (100–1,000 nm) to 5 µm microparticles or microvesicles. Nanovesicles—the EV population most commonly thought of as exosomes—are thought to be derived from the fusion of specialized trafficking multi-vesicular endosomes within the cell membrane (11). In contrast, microvesicles are thought to be pinched or shed from the cell membrane (7). Microvesicles also include vesicles derived from apoptotic cells (ApoEVs) (12–14), which were formerly considered as debris rather than purposefully packaged compartments with immunoregulatory functions. The heterogeneity in nomenclature combined with historical EV isolation techniques that filtered out larger vesicles makes it difficult to compare studies (15). Moreover, some ApoEVs resemble nanovesicles in size, morphology, and surface markers (16). Nonetheless, these differences in biogenesis yield significant information about the host cells as well as the potential purpose of the resulting EVs.

Communication of EVs with immune cells occurs through a variety of mechanisms that are tightly paired with the intended downstream function. For example, EVs can be transmitted *via* direct cell-to-cell contact in the immune synapses, which are junctions between antigen-presenting cells (APCs) and T cells that mediate the antigen presentation process (17–19). EV membranes can include surface molecules that facilitate binding with the extracellular matrix (ECM) (20). The location of EVs within the ECM can also dictate certain physiological processes such as differentiation and angiogenesis (21). During skeletal regeneration, matrix-bound EVs guide macrophage differentiation and downstream myogenesis of skeletal muscle progenitor cells (22, 23). EVs can also be freely shed to travel through interstitial fluid to the lymph nodes or diffuse into the bloodstream (24). Each of these mechanisms becomes important for understanding the development of immune response leading to autoimmune diseases.

Proteomic, genomic, and metabolic data indicate that EVs from distressed cells differ in size, content, quantity, and biogenesis from healthy cells (12, 25). Healthy cells exposed to cellular stress show increases in EV production, content, and uptake. Bovine granulosa cells that were exposed to H_2_O_2_ oxidative stress released exosomes containing anti-oxidative molecules (26). Serum-starved mesenchymal stem cells (MSCs) produces exosomes that traffic to neurons 22-fold more efficently than non-serum deprived MSCs (27). While EV content and production were modulated when endothelial cells were exposed to hypoxic conditions, the properties were not reflected when exposed to high glucose (28). These suggest that EVs can modulate communication of stress signals, although not communicate all stresses equally.

## EV Production, Cellular Stress, and Autoimmunity

In the context of autoimmunity, the relationship between cellular stress and EV biogenesis is especially significant. Cellular stress is implicated in the initiation of many autoimmune diseases (3). While there are genetic factors that contribute to autoimmunity, genotype alone does not explain all the incidences. EV content varies widely based on cell type as well as the amount of cellular stress. It is unclear whether there is a consistent purpose for EV communication in autoimmunity. In some cases, EVs could function as a warning or protective signal to surrounding cells. On the other hand, it could simply be a disposal mechanism gone wrong. For example, ineffective clearing of ApoEVs can result in necrosis, causing the release of autoantigens with other pro-inflammatory signals, thereby contributing to autoimmune disease (12).

EV-mediated intercellular communication has garnered increasing attention and is thought to have a critical role in the development and progression of autoimmunity. In the same manner that tumor-derived EVs can condition bone marrow-derived cells to prepare distal organs for the arrival of metastatic cells, EVs from autoimmune organs can educate the immune system on how to react (24, 29). EVs can carry autoantigens, a “self” protein or protein complex that triggers an immune response, which then facilitates an autoimmune disease **(** **Table 1** **)**.

**Table 1:** Autoantigens found in EVs.

Disease	Autoantigens	Type of EVs	Cell type	Size	Reference
SLE/SS	RNPs Ro/SSA, La/SSB, SM	NV + MP	SGEC; non-neoplastic	50–100 nm	(30)
	G3BP, 14-3-3n, B6 tubulin		Peripheral Blood mononuclear cells	100–1,000 nm	(31–35)
					(36)
Diabetes (T1D)	GAD 65 IA-2	NV	Rat/human islets,	100–150 nm	(37)
	Proinsulin, ERV Gag, Env		MSC insulinoma		(38, 39)
					(40)
Rheumatoid Arthritis	IL-1, Citrillinated Proteins, DEK	NV + MP	Platelets, FLS, leukocytes	30–1,000 nm	(41–45)
Vtiligo	tyrosinase, MART-1	ApoEV	Melanocytes	200+ nm	(46)
Pre-eclampsia	NEP, HLA-DR, HMGBI	MP	Synoytiotrophoblasts	200–1,000 nm	(47–49)

Disease type, autoanitgen found in or on EVs, classification of EVs, cell source, and size of EVs. NV, Nanovesicles; MP, Microparticles; RNP, Ribonucleoproteins; Ro/SSA; Ro protein/Sjögren’s Syndrome related antigen A; La/SSB, La protein/Sjögren’s Syndrome related antigen B; SM, Smith antigen; G3BP, Galectin 3-binding protein; GAD65, Glutamic Acid Decarboxylase- 65K; IA-2, Islet Antigen 2; ERV Gag, Endogenous retrovirus Gag antigen; IL-1, Interleukin 1; NEP, Neprilysin; HLA-DR, Human leukocyte antigen- DR; HMGBI, High Mobility Group Box 1.

The origin of autoimmunity is not known for many diseases, and clinical treatments frequently rely on addressing the symptoms rather than the root cause. For example, the standard treatment for Type 1 Diabetes is delivery of exogenously produced insulin, and rheumatoid arthritis is treated *via* delivery of systemic immunosuppressant drugs such as steroids. The growing literature about EVs’ role in antigen presentation may bring the fields closer to not only explanations of disease initiation, but also methods to diagnose and treat autoimmunity.

### Systemic Lupus Erythematosus (SLE)

**Figure 1:**
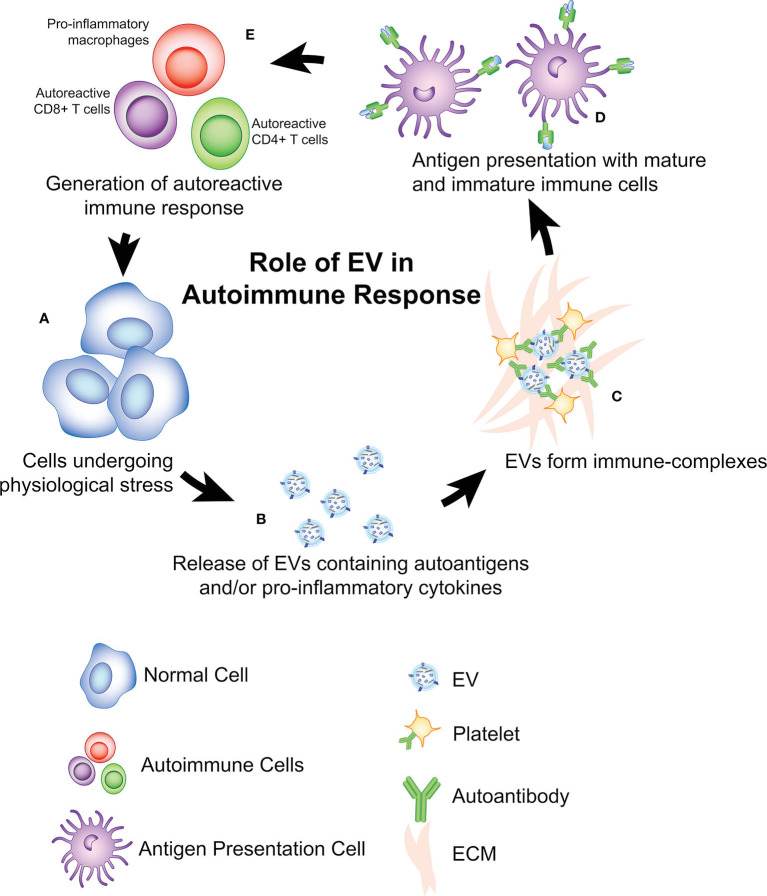
Role of EVs in antigen presentation and initiation of autoimmune diseases. **(A)** a potential theory is that cells undergoing physiological stress initiation autoimmunity via release of **(B)** EVs containing autoantigens, pro-inflammatory cytokines, and/or genetic material (i.e. miRNA, DNA). **(C)** In some cases, these EVs can interact with ECM, antibodies, platelets to form immune-complexes (ICs) that can also facilitate presentation to **(D)** immature and mature antigen presenting cells. After antigen presentation, an adaptive autoimmune response is developed **(E)** as indicated by the expansion of autoreactive CD8+ T Cells, CD4+ T cells, autoreactive B cells, and the pro-inflammatory activation of innate immune cells (i.e. macrophages, neutrophils). This process produces a positive feedback loop, contributing to more cellular damage within the autoimmune microenvironment which leads to the production of more EVs.

One common autoimmune disorder associated with EVs is SLE. SLE is characterized with the hyperproduction of autoantibodies and the accumulation of immune-complexes (ICs), leading to inflammation and tissue damage (**Figure 1**) (31). ICs are formed when EVs containing surface antigens bind to circulating immunoglobulins or ECM proteins, thereby facilitating immune recognition (50, 51). These ICs induce complement-mediated immune responses (52). Apoptosis and immunogenic apoptotic bodies are commonly found in SLE, and may contain dsDNA or other nucleic acids (32, 36). These EVs may stimulate type-1 interferon (IFN) production, which is another major disease contributor in SLE (31). These EVs may also contain autoantigens such as galectin-3-binding protein (G3BP), which is strongly correlated with IC production and increased adherence to the endothelium and basement membrane (32–34).

### Diabetes

Type 1 diabetes (T1D) is a tissue-specific autoimmune disease caused by the autoreactive T cell–mediated destruction of insulin-producing β-cells in the pancreas. EVs are thought to contribute to T1D development in several ways. First, there is a working hypothesis that EVs containing autoantigens are released from stressed cells (**Figure 1**), leading to the activation of autoreactive T cells (40). Autoantigens (ZnT8, insulin, GAD65, and IA-2) have been found in EVs derived from pancreatic islet cells, in both human and rat models (37, 38, 53). These EVs are subsequently endocytosed by dendritic cells (DCs) followed with the activation of the APCs. Aside from the autoantigens, EVs derived from islets also contain immunostimulatory cytokines such as IFN-γ. These cytokines are able to stimulate monocytes, autoreactive B and T cells, which further contribute to the destruction of pancreatic cells (38, 54, 55). In this manner, EVs serve as an autoimmune trigger by facilitating the antigen presentation and activation of autoreactive immune cells (37).

EVs are also involved in the development of Type 2 Diabetes (T2D) by promoting insulin resistance *via* downregulation of glucose transporter type 4 (GLUT4) (56, 57). Microvesicles derived from pro-inflammatory macrophages have been shown to reduce insulin signal transduction and GLUT4 activity (57). Moreover, ApoEVs may induce pro-inflammatory macrophage polarization *via* secretion of mir-155, a potent circulating microRNA (miRNA) that is clinically elevated in T2D studies (58, 59).

### Rheumatoid Arthritis (RA)

RA is a systemic autoimmune disease characterized by chronic inflammation of the synovial joints and the erosion of cartilage (60). EVs are also implicated in RA pathogenesis. Clinically, RA patients have increased EV concentration in synovial fluid and the joint space in comparison to healthy controls (41, 61, 62). The main sources of EVs in RA are platelets, leukocytes and fibroblast-like synoviocytes (FLS) (41–44). Platelet-derived EVs (PEVs) are of particular interest as their higher concentrations in patients with RA and are correlated with disease severity (61, 63). PEVs present interleukin-1 (IL-1) on their surface, which stimulate FLS to release pro-inflammatory IL-6 and IL-8 (44). Stimulated FLSs are also known to release a number of ECM-degrading enzymes that contribute to joint destruction (41). RA-associated EVs also frequently express citrullinated proteins including fibrinogen, fibrin, fibronectin and vimentin which can act as autoantigens (42, 45). EVs containing citrullinated proteins are of particular interest, given that anti-cyclic citrullinated peptide (anti-CCP) antibodies have a 96% specificity for the diagnosis of RA (63). These EVs can also form ICs which are pro-pro-inflammatory and stimulate neutrophils to release leukotrienes, which further perturbate the inflammation in the joint (42).

### Vitiligo

Vitiligo is a disorder consisting of the destruction of melanocytes leading to the depigmentation of the skin (64). Oxidative stress *via* reactive oxygen species (ROS) causes damage to melanocytes which then release antigens through a number of pathways, including EVs (65). When exposed to ROS, melanocytes release EVs containing tyrosinase and Melan-A (MART-1), a melanosome antigen recognized by T cells, thus leading to activation of the autoimmune response (46, 66). Vitiligo also develops as an immune related adverse event (irAEs) following checkpoint inhibitor cancer immunotherapy (67, 68). Interestingly, in the case of melanoma, vitiligo is associated with successful treatment, and individuals with pre-existing vitiligo have lower frequencies of melanoma cancer development (69, 70). This suggests a dynamic relationship between T cell activation and melanocyte destruction.

### Pre-Eclampsia (PE)

PE is a leading cause of maternal death, affecting 2–8% of pregnancies (71, 72). It manifests in two stages, the first being incomplete development of the placenta, while the second stage consists of the main pathology of the disease due to systemic inflammation in the mother (47). It is believed that EVs are directly involved in both stages of PE pathogenesis (73, 74).

During normal pregnancy, exosomes influence the spiral artery remodeling in the uterus, forming the blood vessels that allow for sufficient delivery of nutrients and oxygen from the mother to the fetus (75). Over the course of spiral artery remodeling, uterine arteries expand from 200 μm to 2 mm in diameter. This process occurs through the sequential steps of vessel dilation, vascular smooth muscle cell separation, endothelial cell swelling, extravillous trophoblast (EVT) cell invasion and fibrinoid deposition (75). In early-onset PE, poor placentation occurs due to insufficient spiral artery remodeling. The intrauterine oxygen tension in PE remains at hypoxic levels (1%-3%), leading to a 7-fold increase in exosome release (75, 76). In addition, bioactivity of the exosomes showed increased levels of hypoxia-induced factor 1-alpha (HIF-1α) and IL-8 (75).

The release of these exosomes is dependent upon the oxygen tension within the uterus. During the first trimester, intrauterine oxygen tension as low as 3%. The lower oxygen tension causes an increase in exosomal release and bioactivity, stimulating the invasion of the EVTs to perform proper placentation (75). Cytotrophoblast exosomes regulate this invasion in both a time and dose-dependent manner (75). After adequate spiral artery remodeling occurs, the oxygen tension increases to 8% (76). It has been hypothesized that the increase in exosomal release under hypoxia is an adaptive measure taken by the body to induce the proliferation and invasion of the EVTs to correct the poor placentation while sufficiently remodel the spiral arteries (77). However, the mechansims underlying why this process leads to normal pregnancy in some and PE in others is still unclear.

Much like in other autoimmune diseases summarized in this review, EVs generated *via* cellular stress contribute to PE. *In vitro* studies reveal that human placental cells (BeWo) undergoing endoplasmic reticulum stress (ER-stress) produce EVs that contain damage-associated molecular patterns (DAMPs) thought to contribute to the development of PE (78). EVs from the syncytiotrophoblast, the epithelial covering of the placenta interfacing with maternal blood, shed into the maternal blood carrying antigens (47, 74). EVs collected from patients with PE show significantly higher neprilysin and human leukocyte antigen (HLA-DR), with significantly lower synctin-2 compared to EVs from normal pregnancies (47, 79). Syncytiotropoblast-derived EVs stimulate monocytes, neutrophils, B and T lymphocytes which then release IL-6, IL-8 and tumor necrosis factor-α (TNF-α), leading to a pro-inflammatory response (74).

## EV-Facilitated Therapies to Treat Autoimmune Disease

### MSC-Derived EVs as Autoimmune Diseases Therapies

Mesenchymal stem cells (MSCs) are key sources of immunosuppressive soluble factors that are critical to tissue repair and regeneration (80). Because of their unique function, MSCs or MSC-derived EVs (MSC-EVs) have been therapeutically administered to heal wounds and repair tissue after myocardial infarction (80, 81). Current autoimmune therapeutic strategies involving MSC-EVs have shown efficacy in reducing inflammation in several animal models including experimental autoimmune encephalomyelitis (EAE) mice, which can serve as a model for multiple sclerosis (MS). EAE mice treated with EVs from stimulated MSCs showed reduction in the clinical score, demyelination and neuroinflammation, with increased secretion of anti-inflammatory cytokines as well as upregulated regulatory T cells (82, 83). In a rat experimental autoimmune uveitis (EAU) model, MSC-EVs lead to a reduction of EAU in the eyes (84).

In addition to reducing inflammation, MSCs have been clinically explored to prevent tissue damage and promote regeneration. Tao et al., used human embryonic MSC-EVs to regenerate tissue following osteoarthritis-induced cartilage damage (85). The group found MSC-EVs improved histological scores and gross appearance of the cartilages over controls, with restoration of cartilage as well as subchondral bone by week 12. The characteristics of hyaline cartilage closely resembled the unoperated control. In addition to this, exosomes from human synovial MSC (SMSC) have been shown to promote cartilage regeneration with preventive effect in a rat osteoarthritis model (85). Bone marrow-derived MSC-EVs were shown to delay the inflammatory response in RA, measured through reduction in joint damage and pro-inflammatory gene expression (86). Interestingly, both MSC-EVs classified as exosomes and microvesicles can protect mice from joint damage (87).

Induced pluripotent MSC (iMSCs) are a valuable cell source for EV treatments due to their compatibility with autologous transplantation (88). Moreover, iMSCs pocesses the potential for scalability, which is vital due to the enourmous amounts of cells needed to produce therapeutic quantities of EVs and the limited population of source cells from which to derive. When tested for their ability to promote proliferation in a skin wound healing model, EVs derived from iMSCs performed similarly to EVs derived from native MSCs (89). Comparable studies show similar therapeutic results in osteoarthritis (90).

### DC-Derived EVs as Autoimmune Diseases Therapies

DCs can regulate adaptive immunuity through antigen presentation to T cells and in this manner, DC derived EVs (DC-EVs) have also been explored for treatment of autoimmune diseases. DCs differ from MSCs slightly in that DCs can perform either pro-inflammatory or anti-inflammatory functions (91), thus DC-EVs must be harvested from DCs that have been conditioned to an immunosuppressive phenotype to illicit an anti-inflammatory effect. Nonetheless, DC-EVs have been assessed for their therapeutic potential to treat autoimmune diseases with cheerful efficacy across several animal models. DC-EVs delayed the onset of murine collagenase-induced arthritis (CIA) and dampened the severity of established arthritis (92). Mice that received EVs from engineered bone marrow DCs (BMDCs) even showed a reversal effect of established CIA, with the exosomes having a stronger immunosuppressing effect than the parental DCs (92). DC-EVs have also been studied for Irritable Bowel Disease (IBD), with showing a protective effective through induction of regulatory T cells (93).

### EVs as A Drug Delivery Vehicle

Most of the current EV treatment strategies involve harvesting EVs from stimulated MSCs or DCs **(** **Table 2** **)**. There are fewer studies that load harvested native EVs with therapeutic reagents for treatment in autoimmune disease, which could be a future growth opportunity. Various methods have been explored to utilize EVs to deliver drugs for septic shock, breast cancer, prostate cancer, hepatocarcinoma, and Parkinson’s disease (94–99). EVs can be loaded externally, i.e. drugs are loaded *via* membrane manipluations. Sun et al. mixing the exosomes with curcumin and demonstrated an enhanced anti-inflammatory effect of curcumin in septic shock treatment (97). Loading EVs with aptamers through mechanical extrusion of breast cancer cells enhances breast cancer targeting effect of the EVs (94). Chemotherapy may also be improved with EVs, as co-incubated prostate cancer-derived EVs with paclitaxel showed a stronger therapeutic effect (98) than paclitaxel alone. EVs have also been loaded using internal mechanisms, i.e.loading cells and harvesting the drug-laden EVs from cell culture media. The Batrakova group has successfully used this strategy to delivery nucleic acids in Parkinson’s Disease, cancer, and Batten’s Disease (100–102). Other groups transfect cells with vectors encoding for nanobodies to enhance the EVs targeting capacity (103).

**Table 2:** The use of extracellular vesicles (EVs) in clinical trials related to autoimmune disease.

Source	Type of EVs	Disease/Indication	Trial Number	Purpose
MSC	Exosome, conditioned media	GvHD		Treatment
Umbilical mesenchymal stem cells derived	Exosomes	Dry eye in patients with cGVHD	NCT04213248	Treatment
Umbilical mesenchymal Stem cells derived	Exosome, microvesicle	Type I diabetes	NCT02138331	Treatment
Plasma	Exosomes	Ulcer	NCT02565264	Treatment
Blood, urine	Exosomes	autoimmune thyroid heart disease	NCT03984006	Biomarker
Blood, urine	Exosomes	Adult-onset autoimmune diabetes	NCT03971955	Biomarker
Blood, urine	Exosomes	Type 1 diabetes	NCT04164966	Biomarker
Blood, urine	Exosomes	Relapsing multiple sclerosis (RMS)	NCT04121065	Biomarker
Blood, urine	Exosomes	Systemic autoimmune diseases (broad)	NCT02890121	Biomarker
Blood, urine	Microparticles	Systemic lupus erythematosus and systemic sclerosis	NCT03575156	Biomarker
Urine	Exosomes	Systemic lupus erythematosus	NCT04534647	Biomarker
Blood	Microparticles	Giant cell arteritis	NCT02333708	Biomarker
Blood; platelet	Microparticles	Type 1 diabetes	NCT01397513	Monitoring
Blood, urine	Microparticles	Type 1 diabetes	NCT00934336	Monitoring
Blood	Exosome	Type 1 diabetes	NCT03392441	Monitoring
Blood	EVs	Type 1 diabetes (T1DM), type 2 diabetes	NCT03106246	Biomarker
Blood, urine	Exosome	Connective tissue diseases (CTD) or systemic autoimmune diseases (SADs)	NCT02890147	Biomarker
Blood	Microparticles	Pregnancy related vascular complications	NCT00485784	Biomarker
Blood	Microparticles	Pregnancy related vascular complications	NCT01736826	Biomarker
Urine	Microparticles	Pre-eclampsia	NCT04520048	Monitoring
Blood, urine, and placental	Exosome	Pre-eclampsia	NCT04154332	Biomarker
Blood, umibilical cord mesenchymal stem cells (UCMSCs)	Exosome	Pre-eclampsia	NCT03562715	Biomarker

Results from the clinicaltrials.gov search of EVs for the diagnosis and/or treatment of autoimmune disease. This search included derivative terms such as exosomes, microvesicles, microparticles.

### EV Isolation Methods

The manner in which EVs are isolated and characterized *via* size, morphology, and composition is well developed (8, 10, 104–107). However there is a missing connection between how the field characterizes EVs and their classifications *via* biogenesis. For example, ApoEVs can be as large as 5 µm but also as small as 50 nm (12). In the context of autoimmune diseaases, the isolation and classification of EVs are particularly relevant, considering their diversed contribution to disease initiation and propagation. Various EV isolation methods have been used for autoimmune diseases, with each of them targeting specific EV populations.

Differential centrifugation is arguably the most commonly used technique for EV isolation (96, 108–110). This method usually involves a series of low-speed centrifugation steps to remove the cellular debris, followed by ultracentrifugation to pellet down the sub-micron size vesicles. Following differential centrifugation, density gradient centrifugation may further purify EVs (96, 108, 111). Tris/sucrose/D2O mixture is frequently used as a cushion, which is then topped with the partially isolated EVs. After centrifuging the combination at 100,000 x g for 75 min, the cushion is transferred into a new container and diluted, followed by another ultracentrifugation. For further characterization, ultrafiltration can be used. Ultrafiltration passes the EV suspension through one or a series of membrane filters with defined pore sizes, which separates EVs into subtypes based on sizes (110).

Chromatography is also used to isolate EVs. This includes size-exclusion chromatography where porous beads separate the particles based on the hydrodynamic radius. The fraction containing particles with the size of EVs are selectively collected (112–114). Alternatively, as EVs have negative charges, an anion exchange chromatography isolation protocol has been established. Culture medium containing EVs is passed through a solid-phase monolithic ion exchange column and then eluted with NaCl (115).

Precipitation methods are simple but with potentially compromised performance. Polymer precipitation with polyethylene glycol (PEG) is frequently adopted by the commercial kits to eliminate the usage of ultracentrifugation (116). Adding PEG or other super hydrophilic polymers into the EV-containing samples decreases the solubility of EVs, allowing pellet formation at 1,500 x g. Chemical precipitation includes organic solvents, sodium acetate, and protamine (117). Organic solvents precipitate EVs out of the solution through ion-pairing effect, while sodium acetate disturbs EV hydration and pellets by hydrophobic effect. Protamine, as a positively charged molecule, pellets the EVs in a similar manner, and is removed by gel filtration.

Affinity-based separation is another important approach, particularly because of the capacity to isolate EVs based on subpopulations. Here, antibody, aptamer or other affinity reagents are used to isolate EVs with a specific surface expression profile (118–121). Immunoprecipitation takes advantage of the markers commonly enriched on EV surface. The EV suspension is mixed with magnetic beads coupled with the corresponding antibodies, then isolated with a magnetic field. For total exosome isolation, tetraspanins CD63, CD9, and CD81 are normally used as surface antigen targets (96, 122).

Microfluidics are gaining interests in isolating EVs. Microfluidics isolate EVs from human sera or cell culture media in a lab-on-a-chip fashion (123). Chen et al. demonstrated the concept with herringbone groves in 50-μm-wide channels to increase surface area, while attached anti-CD63 antibodies to the surface *via* biotin-NeutrAvidin conjugation for EV capturing. As CD63 is not ubiquitously expressed in all EV population, Wan et al. first designed a lipid nanoprobe that targets the EV membrane, then incorporated it onto a silica herringbone nanostructure microfluidic device for total EV isolation (124).

### Artificial EV Generation Methods

**Figure 2:**
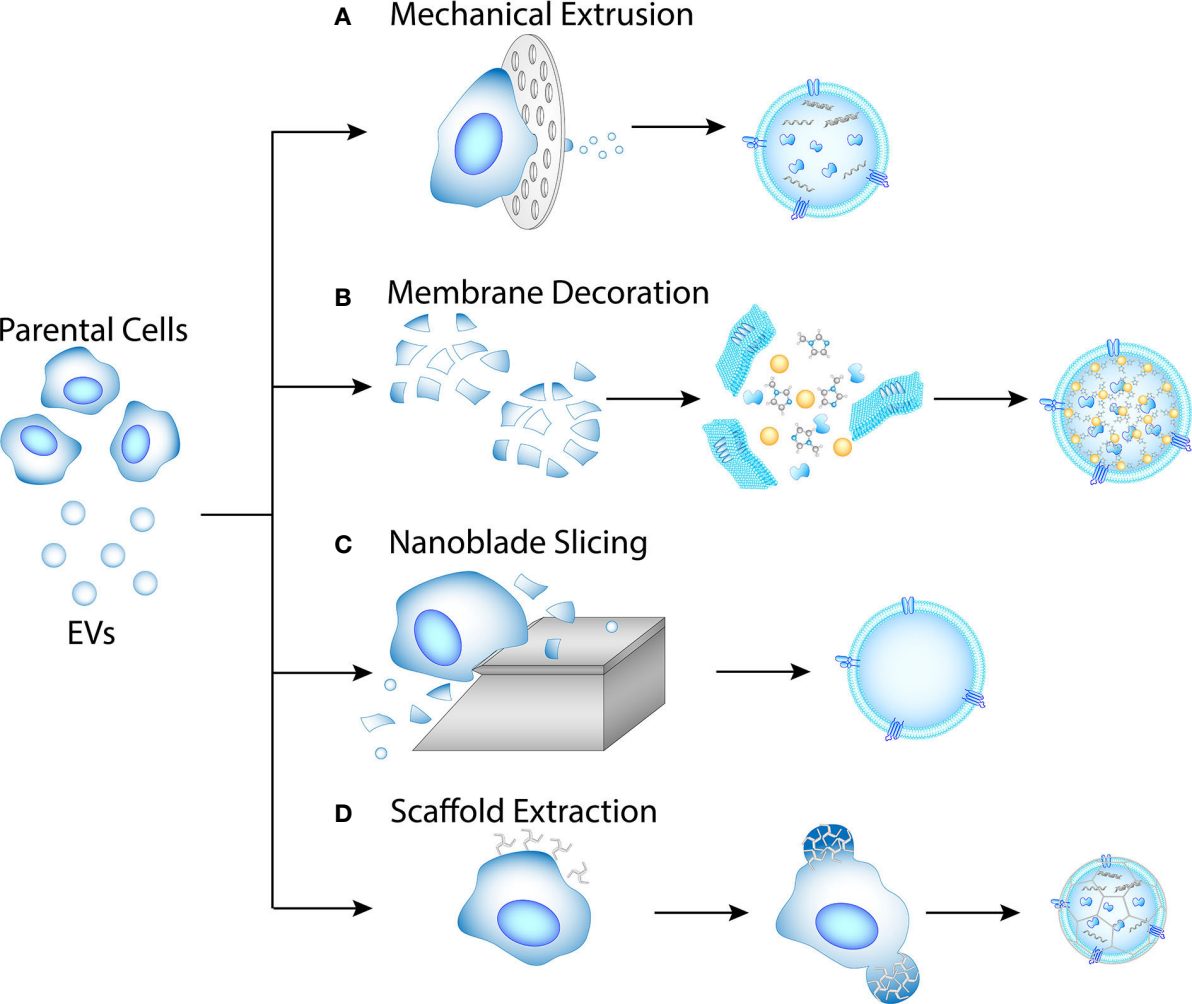
Methods for artificial EV production. **(A)** Mechanical extrusion. Intracellular contents are pertained in the EVs. **(B)** Membrane decoration. Membrane fragments are obtained from lysed cell or EV, then mixed with nanoparticle or MOF cores. **(C)** Nanoblade slicing. Cellular membranes are sliced off the cells and allowed to self-assemble into EVs. **(D)** Scaffold extraction. Clathrin-like nanoparticles may extract EVs from cellular membrane by pinching.

The biocompatibility and functionality of EVs make them an advantageous drug delivery vehicle. However, cells secrete EV at a relatively low rate. Therefore, multiple approaches have been explored to generate EVs artificially **(** **Figure 2** **)**, with a common method being mechanical extrusion **(** **Figure 2A** **)**. Mechanical extrusion methods pass whole cells or membrane lysates through channels of 1–10 μm in diameter (94, 125–127). The channels can be created with tracked-etched membranes or microfluidic devices. Membrane decoration is an efficient method for EV drug loading, which encapsulates a core into lysed cellular or EV membranes **(** **Figure 2B** **)** (95, 128, 129). The core could be a metal-organic framework or nanoparticles.

Some other methods for artificial EV generation that have been investigated include nanoblade slicing **(** **Figure 2C** **)** and scaffold extraction **(** **Figure 2D** **)**. Nanoblade slicing rolls cells over microfabricated 500 nm-thick silicon nitride blades (130). The cellular membrane is sliced into fragments during the process, which then self-assemble into nanovesicles with diameters of 100-300 nm. Substances such as nanobeads can be flowed simultaneously for encapsulation, with an efficiency around 30%. Scaffold extraction is potentially a method for extracting EVs directly from the cellular membrane (131). Using dissipative particle dynamics model, Li et al. simulated the extraction process with clathrin-like nanoparticles, and concluded that the size of the resulting vesicles depends on the concentration of the nanoparticles as wells as membrane surface tension.

### Use of EVs in Clinical Trials for Auto-Immune Diseases

At the time of this writing there were 560 registered trials that involved EVs or derivative terms such as exosomes, microvesicles, and/or microparticles. Of these, 47 trials aligned with autoimmune diseases **(** **Table 2** **)**. A subset of these clinical trials categorize the potential for EVs as autoimmune disease biomarkers. One such study is NCT04164966, an observational study to detect circulating beta-cell exosomes in early onset T1D in a pediatric population. Microparticles are also being studied as a prognostic indicator in SLE (NCT03575156) and arthritis (NCT02333708), highlighting the need for investigation of multiple EV populations. In addition, some trial studies measure EVs concentration as a monitoring tool to quantify the success of their drug intervention. An example of this is NCT01397513, a trial that correlates platelet microparticles to the effect of aspirin dosage on fibrin formation in T1D patients (132).

Of those, there were currently five clinical trials using EVs to treat autoimmune-related diseases. While this seems sparse, it should be noted that these numbers are likely due to the prevalence of cell-based treatments. Of the roughly 10,000 clinical trials related to autoimmune disease, ~300 trials are using MSCs or T cells as a treatment. As the scientific capacity to produce clinical-grade EVs increases, there will likely be an increase in EVs-based therapies for all diseases. As it stands, the results for EVs to treat autoimmune diseases is compelling but limited (**Table 2**). While not classified as such, the clinical manifestation and treatment strategies of graft-versus-host-disease (GvHD) are similar to textbook autoimmune disease (133). Kordelas et al. have demonstrated the feasibility of using the exosome to ameliorate GvHD in one patient that received allogeneic hematopoietic stem cell transplantation (134). Within two weeks, the cutaneous and mucosal GvHD syndromes were remarkably improved, which consequently reduced the dosage of steroid treatment from 125 mg/d to 30 mg/d after exosome therapy. Diarrhea volume of the patient was also objectively reduced.

Interestingly, another clinical trial used two distinct populations of MSC-EVs to treat T1D. The first dose included EVs that were 40–180 nm in size with CD63, CD9, Alix, TSG101 and HSP70 as markers. The second dose consisted of microvesicles of 180-1000 nm in size purified from same MSCs but characterized *via* with annexin V, flotillin-2, selectin, integrin, cd40 and metalloproteinase markers. Unfortunately, the publications listed by the study principal invesetigators do not include data on EVs but rather the delivery of MSCs, making it unclear whether either population of EVs would have had any promising effect (135, 136).

## Conclusion: Future Growth Areas

There are many opportunities for EVs therapies in autoimmune disease. MSC and DC derived EVs are demonstrably efficacious for mounting anti-inflammatory response that promote regeneration, wound-healing, and ameliorating autoimmune disease. However, they are difficult to produce, they require large volumes of cells/media and their batch-to-batch variability lacks the consistency of traditional small molecule therapies. Artificial production of EVs can enable scalable solutions to production, purification, and modification challenges. Currently, the biomarker and diagnostics capabilities of EVs is more advanced than treatments used for autoimmune diseases. New techniques for producing and characterizing artificial EVs could facilitate treatment for autoimmune diseases.

While there has been a large amount of research done on the role of MSC-EVs and DC-EVs, there is comparatively little known about the role of EVs from other immune cells such as macrophages and B cells. Given their role in the pathogenesis of autoimmune disease, it is likely that the EVs from these cells play a significant part in the immune response and could be used in downstream treatment and diagnosis strategies.

Due to the increase of immunotherapy treatments (i.e. checkpoint inhibitors, CAR-T), there will likely be an increase in autoimmune disease events due to irAEs. One example is a treatment using checkpoint inhibitors designed to increase cytotoxic T cell activity in tumors gave rise to cases of vitiligo and hypo/hyper-thyroidism (67, 70). irAEs can range from mild skin reactions to life-long autoimmune-related diseases such as arthritis (137). This convergence in patient populations being treated for irAEs and autoimmune diseases creates an opportunity to gain insight into autoimmune pathology, which is still not universally understood. Investigating EVs in autoimmune disease also presents a unique opportunity to explore bidirectional relationships between diseases. A great example of this are findings that COVID-19 can initiate new-onset diabetes or enhance metabolic complications of pre-existing diabetes (138).

In conclusion, EVs play a significant role in autoimmune disease initiation and progression. Though the role of EVs vary throughout each autoimmune disease summarized, the common themes encourage further investigation of EVs as diagnostic and therapeutic tools. EVs facilitate autoimmune responses by presenting autoantigens from cellularly stressed cells to immune cells. In addition, the EVs can form immune complexes that trigger inflammatory responses. Deciphering the linkage between EVs biogenesis and trafficking may reveal insights into unanswered questions in autoimmune initiation and hold the key to effective treatment.

## Author Contributions

ML, ED, EP, HF, S-YZ, and EW wrote and edited the manuscript. EW conceived and oversaw the project. All authors contributed to the article and approved the submitted version.

## Funding

ML was supported through NIH 7R01CA230339. ED was supported by the Molecular Biophysics and Structural Biology graduate program at the University of Pittsburgh and Carnegie Mellon University.

## Conflict of Interest

The authors declare that the research was conducted in the absence of any commercial or financial relationships that could be construed as a potential conflict of interest.
